# Quantitation of glucose uptake in tumors by dynamic FDG-PET has less glucose bias and lower variability when adjusted for partial saturation of glucose transport

**DOI:** 10.1186/2191-219X-2-6

**Published:** 2012-02-01

**Authors:** Simon-Peter Williams, Judith E Flores-Mercado, Ruediger E Port, Thomas Bengtsson

**Affiliations:** 1 Department of Biomedical Imaging, Genentech, Inc., South San Francisco, CA, 94080, USA; 2 Department of Pharmacokinetics and Pharmacodynamics, Genentech, Inc., South San Francisco, CA, 94080, USA; 3 Department of Biostatistics, Genentech, Inc., South San Francisco, CA, 94080, USA

**Keywords:** variability, glucose correction, MRGluc^MAX^, blood glucose, partial saturation correction, dynamic FDG-PET

## Abstract

**Background:**

A retrospective analysis of estimates of tumor glucose uptake from 1,192 dynamic 2-deoxy-2-(^18^F)fluoro-D-glucose-positron-emission tomography **[**FDG-PET] scans showed strong correlations between blood glucose and both the uptake rate constant [*K*_i_] and the metabolic rate of glucose [MRGluc], hindering the interpretation of PET scans acquired under conditions of altered blood glucose. We sought a method to reduce this glucose bias without increasing the between-subject or test-retest variability and did this by considering that tissue glucose transport is a saturable yet unsaturated process best described as a nonlinear function of glucose levels.

**Methods:**

Patlak-Gjedde analysis was used to compute *K*_i _from 30-min dynamic PET scans in tumor-bearing mice. MRGluc was calculated by factoring in the blood glucose level and a lumped constant equal to unity. Alternatively, we assumed that glucose consumption is saturable according to Michaelis-Menten kinetics and estimated a hypothetical maximum rate of glucose consumption [MRGluc^MAX^] by multiplying *K*_i _and (*K*_M _+ [glucose]), where *K*_M _is a half-saturation Michaelis constant for glucose uptake. Results were computed for 112 separate studies of 8 to 12 scans each; test-retest statistics were measured in a suitable subset of 201 mice.

**Results:**

A *K*_M _value of 130 mg/dL was determined from the data based on minimizing the average correlation between blood glucose and the uptake metric. Using MRGluc^MAX ^resulted in the following benefits compared to using MRGluc: (1) the median correlation with blood glucose was practically zero, and yet (2) the test-retest coefficient of variation [COV] was reduced by 13.4%, and (3) the between-animal COVs were reduced by15.5%. In statistically equivalent terms, achieving the same reduction in between-animal COV while using the traditional MRGluc would require a 40% increase in sample size.

**Conclusions:**

MRGluc appeared to overcorrect tumor FDG data for changing glucose levels. Applying partial saturation correction using MRGluc^MAX ^offered reduced bias, reduced variability, and potentially increased statistical power. We recommend further investigation of MRGluc^MAX ^in quantitative studies of tumor FDG uptake.

## Background

**Figure 1:**
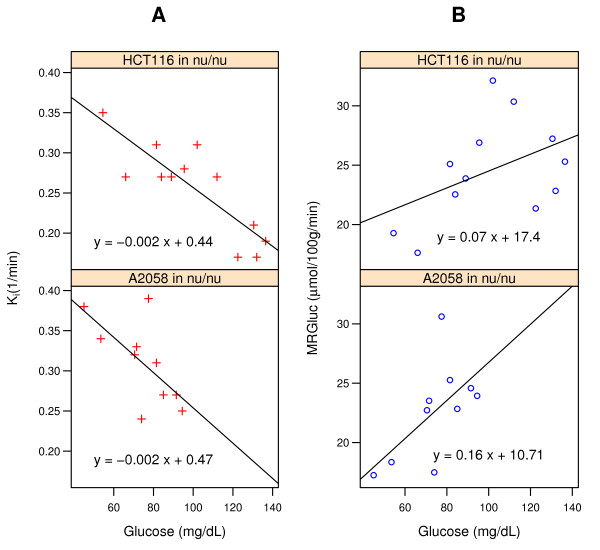
**Relationship between blood glucose concentrations and FDG-PET metrics in two studies**. (**A**) *K*_i _(red), the tracer uptake rate constant from Patlak analysis. (**B**) MRGluc (blue), the product of *K*_i _and blood glucose. The lines represent the linear regression fit to the data.

**Figure 2:**
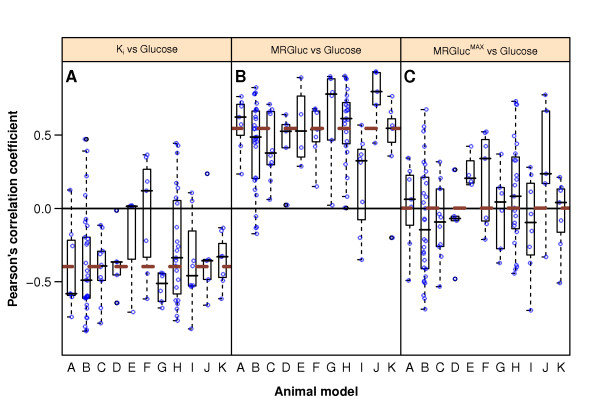
**Pearson's correlation coefficients between blood glucose and *K*_i _(A), MRGluc (B), and MRGluc^MAX ^(C)**. The *y*-axis represents Pearson's correlation value obtained in 112 studies (1,192 observations-see 'Materials and methods' section). The *x*-axis indicates the 11 tumor models (see Table 1). Open circles represent correlation coefficients of individual studies. Box plots show the 25th, 50th, and 75th percentiles of correlation coefficient distributions for every animal model. The thick brown dashed lines show the median for all data.

**Figure 3:**
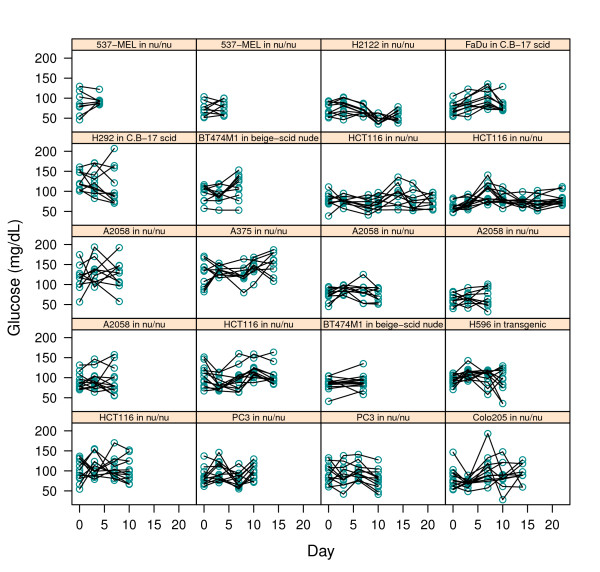
**Individual mouse blood glucose levels (*y*-axis) on multiple scanning days (*x*-axis)**. Mean scan-time glucose was calculated as the average of the pre-scan and post-scan measurements. Each box corresponds to a different cohort of mice as noted.

**Figure 4:**
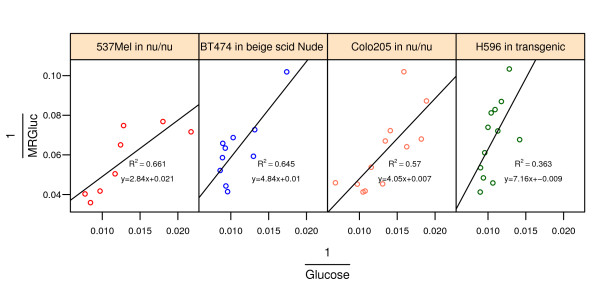
**Diagnostic double-reciprocal Lineweaver-Burk plots of MRGluc versus blood glucose concentration for four different tumor models**. Every point corresponds to an individual mouse. The line represents the linear regression fit to the data.

**Figure 5:**
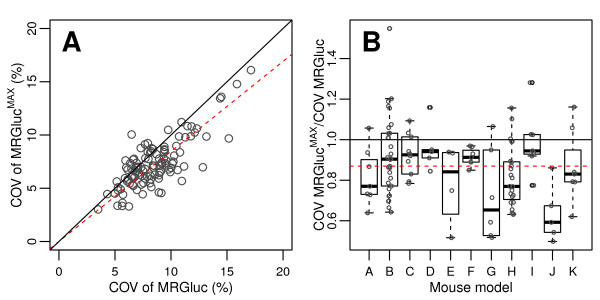
**COVs in 112 FDG studies of 8 to 12 scans**. Each COV was calculated with (MRGluc^MAX^) or without (MRGluc) partial saturation correction. For MRGluc^MAX^, a *K*_M _of 130 mg/dL was used. Each open circle represents the COV calculated for one study. (**A**) Scatter plot of all 112 observations. The solid line represents the identity line. The red dashed line below the identity line represents the linear regression fit to the data. (**B**) Box plots showing the COV ratios grouped according to the 11 mouse models employed. The red dashed horizontal line represents the median of all values.

**Figure 6:**
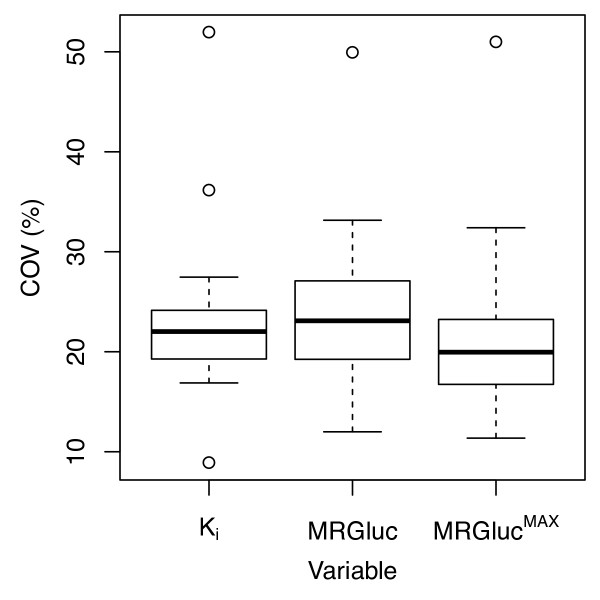
**Percentage test-retest COV**. It was calculated according to Equation 5 for *K*_i_, MRGluc, and MRGluc^MAX ^in 19 studies encompassing 201 mice. Each study consists of 8 to 12 mice scanned at days 0 and 3.

We considered 2-deoxy-2-(^18^F)fluoro-D-glucose-positron-emission tomography [FDG-PET] as a pharmacodynamic marker of antitumor activity during treatments that alter systemic blood glucose levels, for example the Akt inhibitors [1], and sought a metric of tumor glucose uptake that had minimal glucose bias. Inverse correlations of blood glucose with tumor FDG uptake have been demonstrated in multiple settings (see Figures 1 and 2, [2-6]), and this effect was to be expected based on the biochemistry of glucose (and tracer) transport and trapping [7].

We undertook a large series of tumor imaging studies in mice using the metabolic rate of glucose [MRGluc] from Patlak analysis as our preferred estimate of the tumor glucose uptake rate, expecting it to be relatively unbiased with respect to blood glucose. When we undertook a retrospective review of 1,192 such scans performed in study groups of 8 to 12 mice, we observed that our MRGluc data were, in fact, strongly correlated with blood glucose even though individual studies were often underpowered to convincingly show this (see Figure 2B).

We presumed that this correlation caused additional variability in the uptake measurements. Even in the absence of any active treatment, blood glucose levels were not entirely constant in our studies (see Figure 3), so we sought to apply a rational glucose correction to the MRGluc data, noting that the bias reduction benefit must outweigh the cost of the statistical noise introduced by the blood glucose measurements [8].

The original formulations of quantitative glucose uptake measurements using radioactive uptake assays were described comprehensively 35 years ago in the seminal work of Sokoloff et al. [7]. The importance of glucose transport processes based on saturable Michaelis-Menten kinetics has been demonstrated in biochemical studies of glucose transporter 1 [GLUT-1], the dominant glucose transporter in tumors (and erythrocytes and the blood-brain barrier), which have shown that glucose transport into cells can be characterized as a saturable process with a half-maximal-rate Michaelis constant [*K*_M_] of approximately 40 mg/dL [9]; it is the transport step that dominates the overall uptake and trapping rate in many situations [10,11]. Studies in intact animals suggested that the apparent half-saturation constant, *K*_M_, for the GLUT-1-dominated blood-to-brain tissue transport was approximately 5 [12] to 7.3 mM [13], equivalent to 100 to 130 mg/dL.

We reasoned that tissue glucose levels are often neither far below *K*_M _(where the glucose transport rate would be approximately proportional to blood glucose level) nor far above *K*_M _(where the glucose transport rate would be saturated and independent of blood glucose level). Consequently, tissue glucose uptake rates are likely to show an intermediate, nonlinear dependence on blood glucose levels.

We tested this hypothesis using a form of the MRGluc calculation that employs the Michaelis-Menten relationship to compute the hypothetical maximal uptake rate [MRGluc^MAX^] based on an empirical half-saturation *K*_M _of 130 mg/dL (see 'Results' section). This approach should reflect the relatively constant glucose uptake capacity of the tissue rather than the instantaneous uptake rate, more or less independent of variations in blood glucose. We refer to this glucose correction method as *partial saturation correction*.

In this paper, we review 112 separate tumor studies of 8 to 12 dynamic FDG scans each, all analyzed with the Patlak-Gjedde simplified tracer kinetic modeling methods [14-17] yielding the uptake rate constant [*K*_i_] (per second) and the MRGluc (in micromoles per minute per 100 cm^3^). We compared the glucose bias, test-retest, and between-animal variability of *K*_i_, MRGluc, and MRGluc^MAX^.

## Materials and methods

### Data enrollment

The data retrospectively analyzed here came from 11 different xenograft models of human cancers that we have employed in recent projects. Each model is a unique combination of a mouse strain and a tumor line. Included were (1) scans from mice studied at baseline prior to any treatment and (2) any subsequent scans from mice enrolled in control groups not receiving any drug substance. Table 1 describes the 585 mice and 1,192 scans that were included. The mice were studied as cohorts of 8 to 12 individuals. Each member of a cohort had the same gender, age, strain, and tumor type, and they were raised and inoculated at the same time. The average tumor volume in a cohort was 250 to 400 mm^3 ^at the beginning of an imaging experiment. A study is defined here as the imaging of one cohort at one timepoint.

**Table 1:** Data listed by mouse strains and tumor types

Model	Tumor cell line/mouse strain	Tissue of origin	Number of mice	Number of scans
A	BT474MI in beige-scid nude	Breast	44	76
B	HCT116 in athymic nude	Colon	124	339
C	PC3 in athymic nude	Prostate	48	116
D	FaDu in C.B.-17 scid	Pharynx	20	50
E	H292 in C.B.-17 scid	Lung	20	40
F	H596 in huHGF transgenics	Lung	46	81
G	537-MEL in athymic nude	Skin	35	52
H	A2058 in athymic nude	Skin	144	236
I	A375 in athymic nude	Skin	40	75
J	Colo205 in athymic nude	Colon	24	58
K	H2122 in athymic nude	Lung	40	69
	Total		585	1,192

### Imaging

All studies were conducted with the approval of Genentech's AALAC-accredited institutional animal care and use committee. Briefly, animals were fasted overnight with free access to water prior to PET imaging. Sevoflurane in air [18] was used to induce and maintain anesthesia sufficient to restrain the animals while they were scanned prone on the bed of an Inveon MM scanner (Siemens Preclinical Solutions, Knoxville, TN, USA). PET scans lasted 30 min. X-ray CT scans provided attenuation correction. List mode data were typically reconstructed into images with 128 × 128 in-plane voxels of 0.4 × 0.4 mm and 0.8 mm through-plane voxel thickness using vendor-provided iterative OP-MAP implementation with the beta hyperparameter set to 0.05 [19]. The resolution (approximately 1.5 mm), sensitivity, and other performance characteristics of this scanner have been described previously [20]. Body temperature was maintained at 37°C by warm air flows under feedback control. When animals were re-scanned on the second or subsequent days, they were imaged on the same scanner and at the same time of the day as for their first scan. The mice received an FDG tracer dose of approximately 200 μCi by infusion through a tail vein catheter.

### Blood glucose measurements

At every scan, blood glucose measurements were taken twice: once approximately 5 min before and once shortly after the scan approximately 35 min later. The glucose value used in calculations is the mean of the pre- and post-scan measurements. Data were collected with the commercially available Contour glucometer (Bayer Healthcare, Tarrytown, NY, USA). Test-retest reproducibility measurements according to Equation 5 were conducted using this instrument in 20 mice and showed a coefficient of variation of 3.7%.

### Image analysis

Regions of interest [ROIs] were drawn using the image analysis software IRW from Siemens. For any given tumor model, all scans for all animals were analyzed by a single observer following a standard procedure: Tumor ROIs were defined as voxels exceeding a threshold percentage of the maximal tumor signal measured in the last 10 min of the scan; this excluded necrotic or otherwise hypointense regions from the analysis. Mean signal values from the ROIs were used for analysis. Image-derived signal from an ROI in the liver was used as an input function reference region in the Patlak analysis, a technique described in mice by Green et al. [21]. This method is well suited to high-resolution whole-body scans that minimize partial volume artifacts [20,22], such as those used here.

### Time-activity curves and Patlak plots

The Patlak and subsequent statistical analyses were performed with the statistical programming language R [23]. For each tumor model described in Table 1, examples of the time-activity curves and the resultant Patlak plots are presented in Additional file 1 to 11. *K*_i _was measured from Patlak plots of dynamic FDG-PET data [15,16,24]. The linear portion of the plot (beginning approximately 5 min into the time-activity curves) was used for fitting and visually verified: the correlation coefficient *r^2 ^*in each case was at least 0.99.

### Kinetic modeling and partial saturation correction

MRGluc was estimated as *K*_i _× [glucose] × LC, where LC is the lumped kinetic constant (set to unity) and [glucose] is the blood glucose measurement. Some literature denotes this form of MRGluc where LC = 1 as 'MRFDG' [25,26]; we will use 'MRGluc' for its semantic emphasis on glucose (rather than FDG or glutamate) uptake. Although the LC scales the absolute value of the *K*_i _data, it is important to note that the choice of LC has no bearing on the subsequent analysis of glucose correlation, between-animal coefficient of variation [COV], or test-retest reproducibility.

MRGluc and its basic dependence on blood glucose levels were modeled according to Equation 1, a form of the Michaelis-Menten relationship [27,28]:

(1) $$\text{MRGluc}=\frac{\text{MRGlu}{\text{c}}^{\text{MAX}}\left[\text{glucose}\right]}{K_{\text{M}}+\left[\text{glucose}\right]}.$$

MRGluc^MAX ^is the hypothetical maximal value of glucose uptake rate, approached asymptotically as the glucose concentration increases to saturating levels. If it were physically possible, the glucose uptake rate measured along the horizontal asymptote would be expected to show zero correlation with the glucose concentration. The curvature parameter *K*_M _is the Michaelis constant that represents the blood glucose concentration at which the glucose uptake rate is half the maximal (glucose-saturated) rate.

To see if our data plausibly followed the Michaelis-Menten model, we transformed the measurements into the linear double-reciprocal form of Equation 2 and generated the corresponding Lineweaver-Burk plots (some examples are shown in Figure 4):

(2) $$\frac{1}{\text{MRGluc}}=\frac{K_{\text{M}}}{\text{MRGlu}{\text{c}}^{\text{MAX}}}\frac{1}{\left[\text{glucose}\right]}+\frac{1}{\text{MRGlu}{\text{c}}^{\text{MAX}}}.$$

### Computation of MRGluc^MAX^

We divide both sides of Equation 1 by [glucose] and see that

(3) $$K_{\text{i}}=\text{MRGlu}{\text{c}}^{\text{MAX}}\left(\frac{1}{K_{\text{M}}+\left[\text{glucose}\right]}\right).$$

A further rearrangement allows the computation of MRGluc^MAX ^for each individual animal:

(4) $$\text{MRGlu}{\text{c}}^{\text{MAX}}=K_{\text{i}}\left(K_{\text{M}}+\left[\text{glucose}\right]\right).$$

All the data presented in this paper were computed using Equation 4, and group mean data were calculated by sample averaging the results for individual animals within a given study.

### Estimation of *K*_M _by minimizing the correlation between blood glucose and MRGluc^MAX^

We computed estimates of MRGluc^MAX ^with a range of *K*_M _values from 40 to 200 mM and selected the *K*_M _that gave the smallest nonnegative value of the median Pearson's correlation coefficient between MRGluc^MAX ^and [glucose] across all 112 studies. As an exploratory analysis, we also separately estimated *K*_M _for each of the 11 tumor models.

### Variability and reproducibility

Between-animal variability was measured as the COV, calculated as standard error of the estimate divided by the estimate, and expressed as a percentage. Test-retest reproducibility statistics were calculated for 19 studies with the 201 mice that were scanned at day 0 and again at day 3. This was the most common test-retest interval in our data. The COVs were calculated using Equation 5, as described by Weber et al. [29]:

(5) $$\text{CO}{\text{V}}_{\text{study}}=\frac{\overset{\text{i}}{\underset{0}{\sum }}\left|\left(\text{Measuremen}{\text{t}}_{\text{i}\left(\text{baseline}\right)}-\text{Measuremen}{\text{t}}_{\text{i}\left(\text{day}\;3\right)}\right)\right|}{\overset{\text{i}}{\underset{0}{\sum }}\frac{\left(\text{Measuremen}{\text{t}}_{\text{i}\left(\text{baseline}\right)}+\text{Measuremen}{\text{t}}_{\text{i}\left(\text{day}\;3\right)}\right)}{2}}.$$

## Results

### Both *K*_i _and MRGluc are correlated with blood glucose levels

In some studies, correlations between blood glucose levels and the FDG-PET estimates of glucose uptake rate were readily apparent. Two of these are illustrated in Figure 1: panel A for *K*_i _and panel B for MRGluc. As expected, many individual cohorts of 8 to 12 mice were statistically underpowered to show such a relationship.

More important, and remarkable, was the consistent presence and strength of this relationship between blood glucose and tissue uptake rates when seen in the meta-analysis of our large sample of studies. Figure 2 illustrates this using a box plot of Pearson's correlation coefficients between blood glucose and (A) *K*_i_, and (B) MRGluc for all 1,192 scans from the 112 studies. The data are grouped into one box for each of the 11 tumor models, with the median for each box shown as a horizontal line. Data for each tumor model comprised 4 to 30 studies; the open circles within a box show individual studies for full disclosure.

Blood glucose levels were negatively correlated with *K*_i _in 90 studies (Figure 2A). The median correlation coefficient (dashed line) was -0.4. With MRGluc as the metric of tumor glucose uptake rate, 104 studies now showed a positive correlation with a median correlation coefficient of 0.55 (Figure 2B), indicating that factoring in the glucose did not eliminate the bias, but rather changed it from negative to positive.

In this meta-analysis of 112 studies, it is possible to compute for each tumor model confidence interval around the correlation coefficients reported in Figure 2. The statistical methodology and results are presented for the interested reader in Additional file 2.

### Lineweaver-Burk plots

A preliminary analysis of our data simply looked for positive correlations between MRGluc and blood glucose levels in the double-reciprocal Lineweaver-Burk plots that are characteristic of a Michaelis-Menten relationship [27,28]. Thirteen of the first 20 tumor studies we examined had some correlation, judging by eye, encouraging further consideration of the Michaelis-Menten model in our data. It was also apparent that the data were inherently noisy such that individual studies were perhaps underpowered to demonstrate a relationship. No quantitative inferences were drawn from these analyses, however. Four such studies are shown in Figure 4.

### When *K*_M _= 130 mg/dL, MRGluc^MAX ^shows zero glucose bias, on average

Figure 2C shows the correlation between MRGluc^MAX ^and blood glucose when *K*_M _= 130 mg/dL. At this value of *K*_M_, the median correlation coefficient for all 112 studies was practically equal to zero, < 0.0004 (dashed line in Figure 2C). Of the 112 studies, 55 showed a positive correlation, 55 showed a negative correlation, and 2 had practically zero correlation (< 0.003). Increasing values of *K*_M _beyond 130 mg/dL resulted in progressively more negative median correlation coefficients.

### Individual blood glucose often varies between scans

Blood glucose levels recorded at scan time for individual mice on multiple measurement days are presented in Figure 3. Each box contains a different cohort of mice studied on multiple days. Differences in group means and fluctuations over time are apparent despite consistency of handling.

### Between-animal variability

From Figure 5A, we observe that there was typically a reduction in the COV of MRGluc^MAX ^with respect to the COV of the same scans quantified using MRGluc. Most of the points lie below the identity line; 87 of the 112 studies analyzed showed some improvement. The average reduction in COV was measured as 15.5% from the value of the fitted regression line slope of 0.845 shown as the dashed line in Figure 5A.

Our hypothesis was that we could reduce variability by extrapolating the tumor glucose uptake rate measurement to a hypothetical asymptote where glucose is under saturating conditions, and our data seem to support this. Mathematically, the improvement in COV appears to come from the fact that MRGluc^MAX ^is greater than MRGluc by definition. Specifically, because *K*_M _= 130 mg/dL and the glucose measurements are near 100 mg/dL, we observe that, on the average, MRGluc^MAX ^values approximately double those of MRGluc. The standard error in MRGluc^MAX ^is also greater than that in MRGluc, but proportionately less so, and so we get an overall reduction in COV of 15.5%.

### Power estimation and sample size calculation

An overall reduction in COV of 15.5% could translate (statistically equivalently) into either a need for fewer subjects per study or into the ability to detect smaller effect sizes. Generally speaking, the standard error of sample means (and of maximum likelihood estimators in general) is inversely proportional to the square root of the sample size: this implies that to achieve the same between-animal COV when using MRGluc would require an increased sample size of 40% (i.e., 100 × 1/(1-0.155)^2^) compared when using MRGluc^MAX^.

### Test-retest reproducibility

For the 19 studies examined, the median test-retest reproducibility COV results were 22.0% for *K*_i_, 23.1% for MRGluc, and 20.0% for MRGluc^MAX^. Figure 6 illustrates the distribution of COV values for each of the three PET metrics.

### Sensitivity analysis for the between-animal COV as a function of *K*_M_

Varying the value of *K*_M _in the range of 40 to 200 mg/dL did not change the nature of the results: MRGluc^MAX ^gave lower between-animal variability than MRGluc. The reduction in the average COV/*K*_M _correspondence was 10% (40 mg/dL), 15% (100 mg/dL), 15.5% (130 mg/dL), and 16% (200 mg/dL).

## Discussion

### Correlations between blood glucose levels and MRGluc

Figure 2B shows that, in our setting with anesthetized mice, there is undoubtedly a strong and persistent positive correlation between blood glucose and MRGluc across a variety of tumor models and mouse strains. It is possible to calculate confidence intervals for the correlation coefficients; these reinforce our conclusions since only one of eleven models had a 95% confidence interval that included zero (-0.01 to 0.42). These calculations and results are presented in Additional file 2 for the interested reader.

### Use and applicability of MRGluc

Rigorous methods for estimating the MRGluc utilization were developed over 30 years ago and continue to be successfully applied [7,12,26,30,31], not least in tumors [2,4,17,32-36]. However, capturing the rate of glucose uptake in the instant of the scan leads to MRGluc reflecting changes in blood glucose whether or not they are functionally significant to the tissue. For malignant tumors, which are highly glucose-addicted, glucose uptake *capacity *may well be a more important tissue characteristic to consider than the glucose uptake *rate*. MRGluc^MAX ^reduces glucose bias by emphasizing capacity rather than rate.

### Fundamental problem with nonlinear regression estimates of *K*_M _and MRGluc^MAX^

It may be surprising to some readers that we do not employ a nonlinear regression model to simultaneously estimate MRGluc^MAX ^and *K*_M _from measurements of *K*_i _and [glucose]. Although considerable care must be taken, this approach is known to work [37-39] for enzymatic data collected *in vitro *with minimal statistical noise in the measurements. However, it proved to be impossible with our data from living subjects: the objective function was difficult to optimize and subject to very large estimation errors. Mathematically, this is due to maximum likelihood estimates of *K*_M _and MRGluc^MAX ^being highly linearly codependent, and it requires a wide range of glucose values to confidently distinguish the effects of changing *K*_M _and changing MRGluc^MAX^, at least when faced with relatively noisy real-world *K*_i _measurements. This argument is presented in Appendix 1 for the interested reader along with simulations.

### Use of a fixed value of *K*_M_

The use of an apparent *K*_M _value derived in separate experiments and used within a physiologically reasonable range has the mathematical advantage that it reduces the number of parameters we need to estimate from the scan data and thus avoids the use of underpowered determinations made on a case-by-case basis. We used a large sample of studies to determine the *K*_M _at which there was, on average, no net correlation between MRGluc^MAX ^and blood glucose levels (*K*_M _= 130 mg/dL). We regard this as an upper limit; the lower limit might be set by studies on isolated cells where the measurement can be made with full knowledge of the extracellular glucose concentration (*K*_M _= 40 mg/dL, [9]).

A biological advantage is that we are better able to fix the *K*_M _as a constant property of a certain tissue or tumor type under given conditions which are largely dictated by the discrete nature of the molecular determinants, such as the isotype of the glucose transporter, GLUT-1 versus GLUT-3, for example.

### The importance of tissue glucose and blood glucose

Of particular importance should be how the (mechanistically relevant) tissue glucose relates to the (conveniently measured) blood glucose levels [40]. Ideally, we should know the interstitial glucose concentration in the tumor microenvironment, and while the relationship between blood and tissue glucose is of intense interest and active study, it is still not trivial to measure [41-43]. For normal tissues, interstitial glucose may be modeled, but in tumors with all their heterogeneity and variability, this is likely to remain challenging, and this will likely continue to present a significant source of variability in data that depend on tissue glucose but measure blood glucose.

In some tumors, the glucose utilization is so great and the perfusion so poor that the true tissue glucose may be close to zero [44], leaving tissue glucose transport far from being saturated yet also decoupled from blood glucose levels. We have seen from our data is possible, but not typical. More common are cases where the FDG uptake rate does correlate with blood glucose levels, implying some degree of saturation and thus nonzero tissue glucose levels.

### Mathematical expectation of a correlation between *K*_i _× [glucose] and [glucose]

Here, we report an empirical correlation between glucose as it is commonly measured (in the blood) and MRGluc as it is commonly defined and described in the literature (*K*_i _× [glucose], based on blood glucose measurements). Although it is not widely remarked upon in the literature, this correlation appears to be almost inevitable, a natural consequence of the relationship between *K*_i_, MRGluc, and [glucose]. Given the widely described result that tissue FDG uptake rates (*K*_i_) and FDG uptake levels (standardized uptake value [SUV]) are affected by [glucose] [2-6,45-48], truly incredible circumstances must prevail to have zero correlation between MRGlu and [glucose] under all circumstances. A more extensive mathematical analysis of this problem is presented for the interested reader in the Appendix 2.

### Applicability of *K*_M _values across multiple tumor types

Model-specific *K*_M _values might be expected to have some benefit and were tested as an exploratory measure. They made it possible to bring the blood glucose correlation with MRGluc^MAX ^close to zero for each tumor model independently. However, there was no additional improvement in the between-animal variability. Employing a global value of 130 mg/dL seemed adequate for these exploratory studies given that the benefits of using MRGluc^MAX ^are not critically dependent on using a precise value of *K*_M_.

### Alternative linear regression method for estimation of MRGluc^MAX ^with a fixed *K*_M_

Having adopted the use of a fixed value for *K*_M_, we note that a least-squares linear regression method to compute MRGluc^MAX ^is readily apparent from Equation 3 by plotting *K*_i _as a function of (1/(*K*_M _+ [glucose]), giving a straight line with MRGluc^MAX ^as the slope when the regression line is forced through the origin. Estimation of the group mean by linear regression may perform better than averaging individual values (*c.f*., Section II.5 in the book by Christensen [49]). However, when we tested this alternative calculation, we found that it made no appreciable difference to the results. We note that linear regression should offer the greatest benefit where the data contain a wide spread of glucose concentrations; as we noted above, this is not the case for our living-subject data with its relatively narrow range of physiological blood glucose values and relatively high noise level.

### Variability and statistical power of MRGluc^MAX ^compared to MRGluc

As noted, a 40% increase in sample size would be required to achieve a 15.5% reduction in COV. However, translating a reduction in COV to improve statistical power requires additional assumptions, e.g., regarding the potential treatment effect [50]. To make a preliminary estimate, we assume that the relative treatment effect is the same for MRGluc^MAX ^and MRGluc when expressed as a percentage change from baseline (a conservative assumption since MRGluc^MAX ^is an asymptote). In this case, the reduction in the required sample size while maintaining the same error rates (i.e., the same statistical power) is 28.6% (equal to 100 × (1-(1-0.155)^2^)) (see Equation 2 in van Belle and Martin [50]). The actual sample size savings achieved in practice are likely to be smaller than this because assumptions will not hold exactly. In particular, glucose uptake is only approximated by Michaelis-Menten kinetics; *K*_M _is not known exactly; and the error distribution may be neither Gaussian nor perfectly homoscedastic.

No doubt there are many sources of physiological noise contributing to the total observed variability in *K*_i _[51], and blood glucose may be only a small part of that. Nevertheless, a 15.5% reduction in between-animal COV is not trivial and could well become important over the course of many studies or in marginal cases. Also, this improvement should not be considered in isolation, but seen as one step in the evolution of PET methodology over the years.

### Glucose normalization and bias

Although biologically appealing, mixed results have come from previous studies of linear glucose normalizations applied to FDG-PET data [3-5]. Multiplying *K*_i _by blood glucose (or normalized glucose, i.e., [glucose]/100 mg/dL) did not eliminate bias in our data. Some have found that this normalization actually increased variability and was unhelpful [8,52,53], possibly because of the noise introduced by the glucose assay. However, glucose bias was significantly reduced with the nonlinear MRGluc^MAX ^function, while simultaneously achieving reductions in between-animal and test-retest COVs compared to both *K*_i _and MRGluc. This is very encouraging and warrants further investigation.

### Other nonlinear glucose corrections

Given the significant biological noise that remains in FDG-PET data even after various corrections are applied, other line equations that approximate the Michaelis-Menten equation should fit the data and give broadly similar bias reductions and improvements in glucose-derived variability. For example, Wong et al. have demonstrated that using a square-root function of the glucose concentration allowed their clinical SUV data to better classify indolent and aggressive lymphomas [54]. They also suggested, referring to Langen et al. [2], that this correction would not be necessary with dynamic scans quantified with MRGluc.

### Applicability to SUV data

Our preliminary observations confirm that tumor SUV values correlate highly with our *K*_i _data, showing a negative correlation with blood glucose across hundreds of mice and dozens of studies. However, our SUV values were derived from time-activity curves at no more than 30 min after injection of FDG, and with only 5 min of acquisition time, making them statistically noisy compared to purposeful SUV data. We expect that partial saturation correction will have similar benefits with SUV data, but more appropriate experimental data will be required before this can be properly explored. However, applying the square root of glucose SUV correction of Wong et al. [54] to our tumor data did reduce glucose bias and variability compared to MRGluc, almost as much as MRGluc^MAX^. The converse should also be true, suggesting that multiplying SUV by (*K*_M _+ [glucose]) would be effective in the clinical lymphoma setting, while the mechanistic foundation of this correction may make it possible to rationally optimize *K*_M _in different tissues or tumor types.

### Outliers and blood glucose changes during the scan

Individual outliers often exhibited large differences between their pre-scan and post-scan blood glucose levels. We tested some exclusion criteria which censored out data from scans where there had been a 75% or greater change in blood glucose level during the course of the scan. This helped reduce the between-animal variability in some studies. However, a more attractive alternative may be to track and account for the changes in blood glucose occurring during a scan as proposed by Dunn et al. [55]. It would be interesting to evaluate a combination of partial saturation correction and the method of Dunn et al. [55] to better account for both between-scan and intra-scan blood glucose changes.

## Conclusions

Measured in a very large sample of 1,192 nonclinical dynamic FDG-PET scans, it was clear that the rate of tumor glucose uptake estimated by MRGluc was, in most studies, positively correlated with blood glucose levels. This gave an unwanted bias and additional variability in our estimates of tumor glucose uptake rates.

By assuming a Michaelis-Menten relationship, the simple use of *K*_M _+ [glucose] in place of [glucose] as the glucose correction factor had several benefits: the hypothetical glucose-saturated MRGluc^MAX ^was less correlated with blood glucose, had lower between-animal variability, and had lower test-retest variability compared to MRGluc.

## Future directions

This reduced bias and reduced variability may translate into a significant reduction in sample size (up to 28%) for nonclinical treatment studies. Further performance comparisons of MRGluc and MRGluc^MAX ^applied to detect confirmed treatment responses in our nonclinical tumor models have been completed and will be described separately.

It will be very interesting, and straightforward, to see if these findings can be translated to studies of clinical trial data where saturation-corrected SUV data could be calculated by multiplying SUV by (100 mg/dL + [glucose]), rather than the more commonly reported glucose-normalized SUV employing ([glucose]/100 mg/dL). In the clinical trial setting, even modest reductions in variability can translate to tangible savings in money, time, and patient enrollment.

## Appendix 1

### On the problem of linearly dependent ML estimates of *K*_m _and *V*_max _from noisy Michaelis-Menten observations

If an adequate probabilistic framework can be specified for a sample data set, maximum likelihood [ML] typically provides an efficient approach for parameter estimation. Equivalently, for data which are conditionally Gaussian-distributed, one may also use nonlinear least squares. However, due to the functional form of the Michaelis-Menten [MM] relationship, *K*_m _and *V*_max _are not uniquely estimable (from each other) from noisy MM observations. This problem is further exacerbated when the MM process is observed in a narrow glucose range (as is the case in our work). The problem can be understood by studying the information matrix for *K*_m _and *V*_max_, but we use a first-order expansion of the ML score function to heuristically verify that the ML parameter estimates of *K*_m _and *V*_max _are strongly co-linear.

Let the true values of *K*_m _and *V*_max _be given by *K*_o _and *V*_o_, respectively. For *j *= 1,...,*n*, let $K_{\text{i}}^{j}=V_{\text{o}}/\left(K_{\text{o}}+{\left[glc\right]}^{j}\right)+\varepsilon^{j}$ be the *j*:th observed rate constant where *ε^j ^*is independently sampled from a zero-mean Gaussian distribution with standard deviation σ. Let $S\left(V_{\max},K_{\text{m}},\sigma \right)=\sigma^{-2}\sum_{j}{\left(K_{\text{i}}^{j}-V_{\max}/\left(K_{\text{m}}+{\left[glc\right]}^{j}\right)\right)}^{2}$ be the ML score function to be minimized. The ML estimates are given by

$$\left\{{\hat{V}}_{\max},{\hat{K}}_{\text{m}},\hat{\sigma }\right\}=\underset{V_{\max},K_{\text{m}},\sigma }{\min}S\left(V_{\max},K_{\text{m}},\sigma \right).$$

We note that estimation of σ does not affect estimates of *V*_max _and *K*_m_, and its consideration is henceforth eschewed.

For reasonably large sample sizes, at convergence, the ML estimates ${\hat{V}}_{\text{max}},{\hat{K}}_{\text{m}}$ satisfy

$$\begin{array}{c} S\left({\hat{V}}_{\text{max}},{\hat{K}}_{\text{m}}\right)\approx \underset{j}{\sum }\left({\left(\varepsilon^{j}\right)}^{2}-2\varepsilon^{j}/\left(K_{\text{o}}+{\left[glc\right]}^{j}\right)[({\hat{V}}_{\text{max}}-V_{\text{o}})-V_{\text{o}}/\left(K_{\text{o}}+{\left[glc\right]}^{j}\right)\left({\hat{K}}_{\text{m}}-K_{\text{o}}\right)\right]\\ \;\;\;\;\;+1/{\left(K_{\text{o}}+{\left[glc\right]}^{j}\right)}^{2}{\left[\left({\hat{V}}_{\text{max}}-V_{\text{o}}\right)-V_{\text{o}}/\left(K_{\text{o}}+{\left[glc\right]}^{j}\right)\left({\hat{K}}_{\text{m}}-K_{\text{o}}\right)\right]}^{2}. \end{array}$$

Taking expectations over the noise process, *ε^j ^*yields that on the average, the score is minimized approximately when $\left({\hat{V}}_{\text{max}}-V_{\text{o}}\right)=V_{\text{o}}/\left(K_{\text{o}}+\left[m.glc\right]\right)\left({\hat{K}}_{\text{m}}-K_{\text{o}}\right)$, where [*m.glc*] represents the mean glucose measurement. The result holds when the glucose measurements have low spread but can be shown to hold approximately even as the spread around [*m.glc*] increases. Thus, ${\hat{V}}_{\text{max}}$ is linear in ${\hat{K}}_{\text{m}}$ with a slope equal to *V*_o_/(*K*_o_+[*m.glc*]).

**Figure 7:**
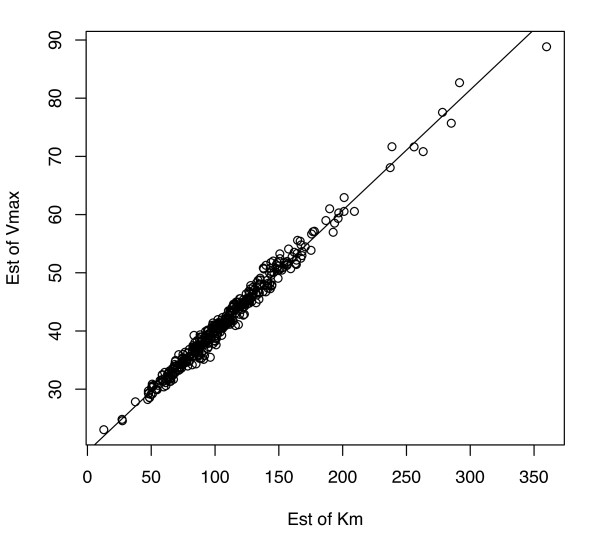
**Highly linearly dependent ML estimates of ${\hat{K}}_{\text{m}}$ and ${\hat{V}}_{\text{max}}$ from perfect model simulations with {*K*_m _= 100, *V*_max _= 40, σ = .025}**.

To illustrate, we ran 400 simulations with the following parameters: *V*_max _= *V*_o _= 40, *K*_m _= *K*_o _= 100, σ = .025, where glucose was randomly sampled from a Gaussian distribution with the mean [*m.glc*] = 100 and standard deviation of 20. Each such simulation used a total of *n *= 20 observations. These parameter settings were chosen to simulate data which closely mimics the previously presented data. The R function *nls*() was used for ML estimation. Figure 7 shows pairs of estimates of *V*_max _(i.e., ${\hat{V}}_{\text{max}}$ on the *y*-axis) and *K*_m _(${\hat{K}}_{\text{m}}$ on the *x*-axis) from these 400 simulations. As can be seen, the ML estimates are highly linearly dependent and have a slope of 0.2072, very near to that derived by *V*_o_/(*K*_o_+[*m.glc*]) = .20. Further, the sample correlation in this plot is .995, indicating that estimates are not uniquely identifiable from the data.

**Figure 8:**
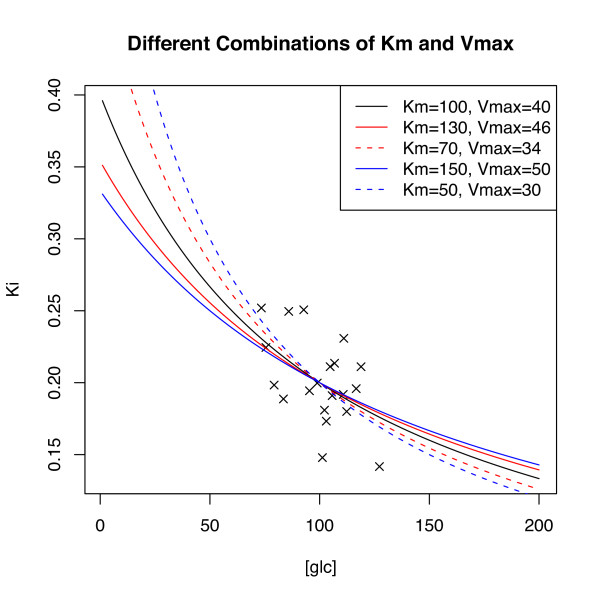
**Plotted sample data can be expected to fit any of the five depicted MM models**.

Although slightly improved, simulations verify that the above problem persists even as the spread in [*glc*] is greatly increased. Figure 8 sheds some intuition on the overall problem. Along with the black MM curve used to generate 20 sample data points (denoted by black 'x') are the MM curves for four other parameter settings (see figure legend for parameter values). As is evident, the sample data cannot be expected to 'choose' efficiently among the depicted candidate models as increases in *K*_m _are 'traded' for increases (and vice versa) in *V*_max _at the rate of .2. This estimation issue remains even as the sample size is increased albeit with decreasing overall sample variability at a rate of 1/*sqrt*(*n*).

## Appendix 2

### On the correlation between *MRgluc *and [*glc*]

As noted, several authors have described studies where the observed rates *K*_i _were negatively correlated with the glucose measurements [*glc*]. We show that when such a negative correlation exists, the correlation between *MRgluc*, defined as the product between *K*_i _and [*glc*], and [*glc*] cannot uniformly equal zero. Indeed, lack of correlation occurs but in a few special cases.

Since *K*_i _> 0 and [*glc*] > 0 are negatively correlated, there exist constants *α *> 0 and *β *< 0 such that the form *K*_i _≈ *α + β*[*glc*] + *ε *describes the correlation between *K*_i _and [*glc*]. Here, *ε *is a zero-mean error process independent of [*glc*] with variance $\sigma_{\varepsilon }^{2}$. Then, with $\mu_{g}^{k}$ denoting the *k*:th raw moment of [*glc*] and with $\sigma_{g}^{2}$ its variance, straightforward algebra shows that the covariance between *MRgluc *and [*glc*] equals

$$cov\left(MRgluc,\left[glc\right]\right)=\alpha \sigma_{g}^{2}+\beta \left(\mu_{g}^{3}-\mu_{g}^{2}\mu_{g}\right).$$

Thus, for *cov*(*MRgluc*, [*glc*]) = 0, we must have $\alpha \sigma_{g}^{2}=-\beta \left(\mu_{g}^{3}-\mu_{g}^{2}\mu_{g}\right)$ or $\alpha /\beta =\left(\mu_{g}^{2}\mu_{g}-\mu_{g}^{3}\right)/\sigma_{g}^{2}.$ This equality can clearly not hold uniformly across a broad range of realistic parameter choices of $\left\{\alpha ,\beta ,\mu_{g},\mu_{g}^{2},\mu_{g}^{3}\right\}$.

We illustrate the result by simulations. Our setting assumes that *K*_i _follows the MM form with constants *K*_m _and *V*_max _and that the observed rate is corrupted by noise. That is, *K*_i _= *V*_max_/(*K*_m _+ [*glc*]) + *ε*, where *ε *is the random Gaussian with zero-mean and standard deviation σ. As is common, we further assume that the rate constant is observed (sampled) in a glucose range between 60 and 140. We note that when *K*_i _is observed in a limited range around some glucose midpoint [*m.glc*], *K*_i _≈ (*V*_max_/(*K*_m _+ [*m.glc*])+([*m.glc*]*V*_max_)/(*K*_m _+ [*m.glc*])^2^)-*V*_max_/(*K*_m _+ [*m.glc*])^2^[*glc*] + *ε*, i.e., *K*_i _is approximately linear in [*glc*].

**Figure 9:**
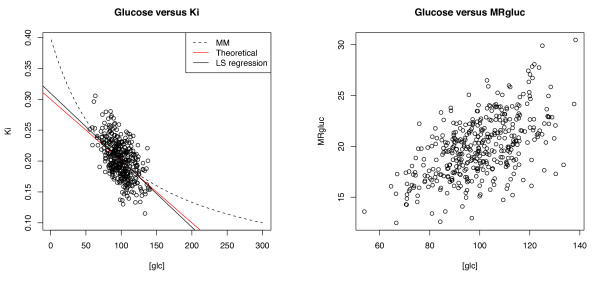
**Scatter plots of [*glc*] vs. *K*_i _(left) and [*glc*] vs. *MRgluc *(right)**. The left panel also shows the underlying MM process (dashed black line) from which the data was sampled, along with theoretical (red solid) and fitted (black solid) regression lines.

The left panel in Figure 9 shows 400 simulated observations drawn from a MM model with *V*_max _= 40, *K*_m _= 100, σ = .025, where glucose was randomly sampled from a Gaussian distribution with a mean of 100 and a standard deviation of 15. As can be seen, in the sampled range, *K*_i _is approximately linear in [*glc*]. The right panel shows a scatter plot of [*glc*] vs. *MRgluc*. Consistent with our derivations, the sample correlations in the two plots are -.48 and .53, respectively. For the chosen parameter choices and glucose distribution, based on the above arguments, the sample correlation between [*glc*] and *MRgluc *should be near to its theoretically predicted value of .51. (For this data, the sample correlation between [*glc*] and *MRgluc^MAX ^*= *K*_i_(*K*_m _+ [*glc*]) is .01.)

## Competing interests

The authors declare that they have no competing interests.

## Authors' contributions

S-PW designed the studies and wrote the manuscript, JEF-M programmed the data analyses and prepared the figures, REP guided the discussion, and TB guided the data analysis and statistics. All authors read and approved the final manuscript.

## Supplementary Material

Additional file 1 **ROI data and corresponding Patlak plots from FDG-PET scans in each of the 11 tumor models A to K discussed in the text (see Table **1**)**. In each plot, the data from one cohort (*n *= 14 to 36) of essentially identical mice are superimposed. Left, in red: the liver-derived input function; center, in blue: the tumor; right, in gray: the Patlak plot. Click here for file

Additional file 2 **Confidence intervals for correlations between PET metrics and blood glucose**. To obtain the 95% confidence limits for Pearson's correlation coefficient (*r*), the Fisher transformation was applied to the sample correlation coefficients. Click here for file
