# Supplementary material for: Quantitation of glucose uptake in tumors by dynamic FDG-PET has less glucose bias and lower variability when adjusted for partial saturation of glucose transport
Source: EJNMMI Res. 2012 Feb 1;2:6. doi: 10.1186/2191-219X-2-6 (PMC3395842; doi:10.1186/2191-219X-2-6)

Model A. BT474M1 breast cancer cells in beige scid nude mice

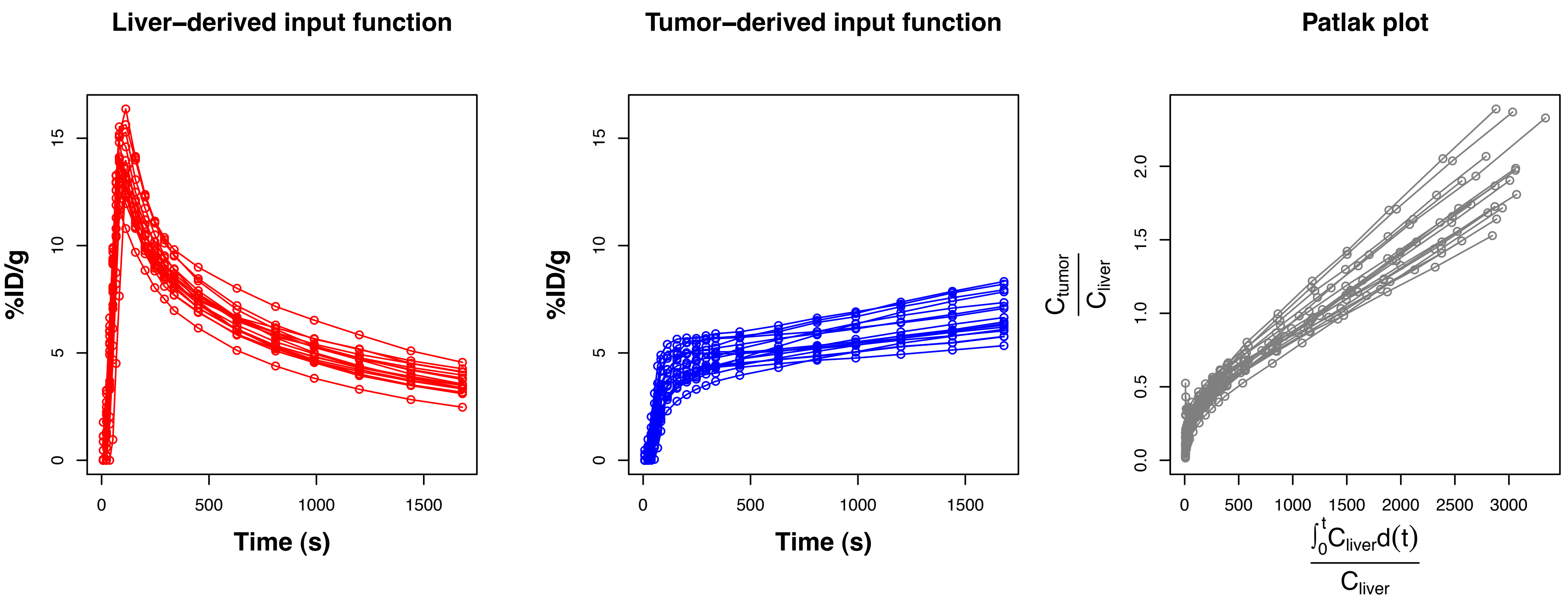

Model B. HCT116 colon cancer cells in athymic nude mice

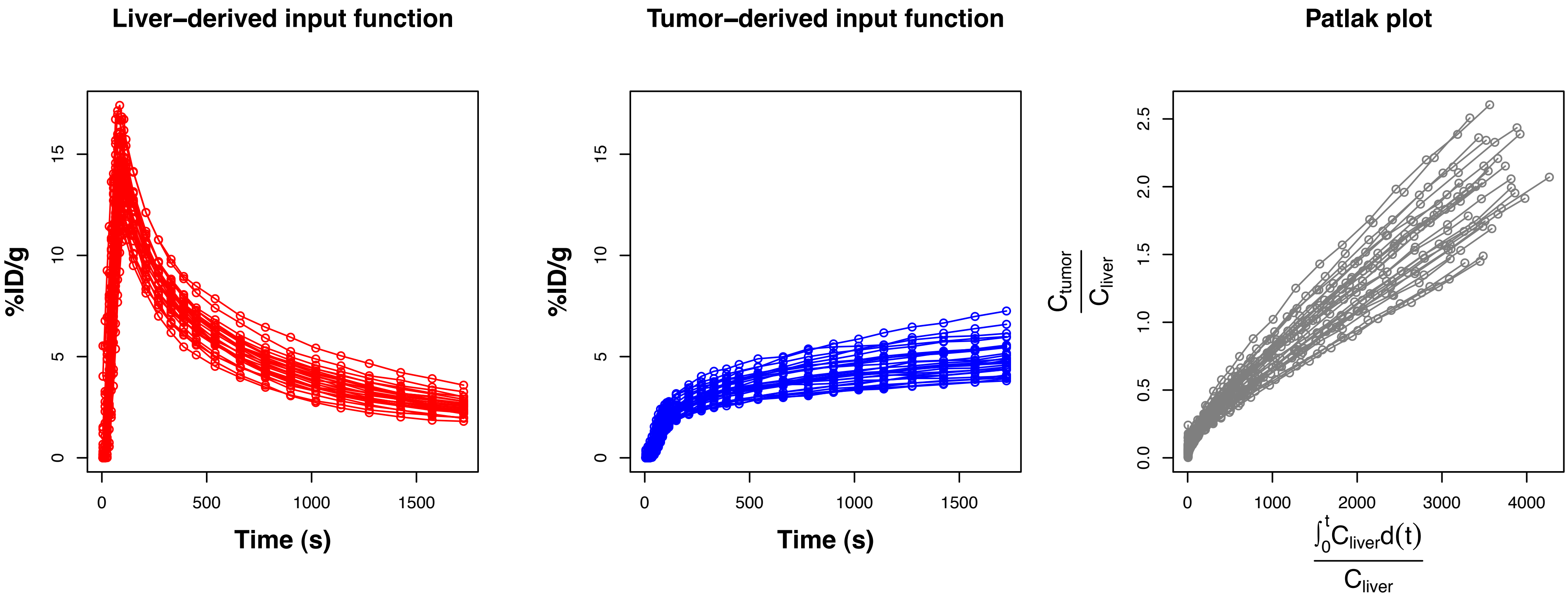

Model C. PC3 prostate cancer cells in athymic nude mice

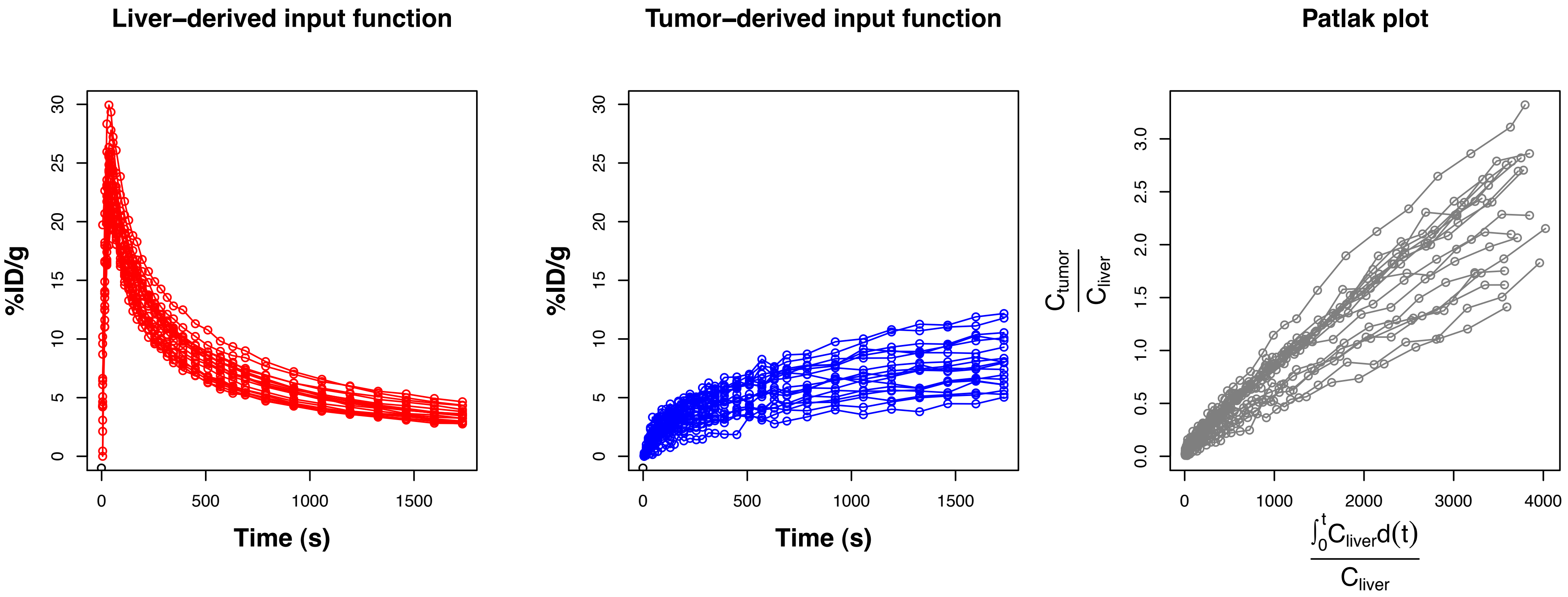

Model D. FaDu pharynx cancer cells in C.B−17 scid mice

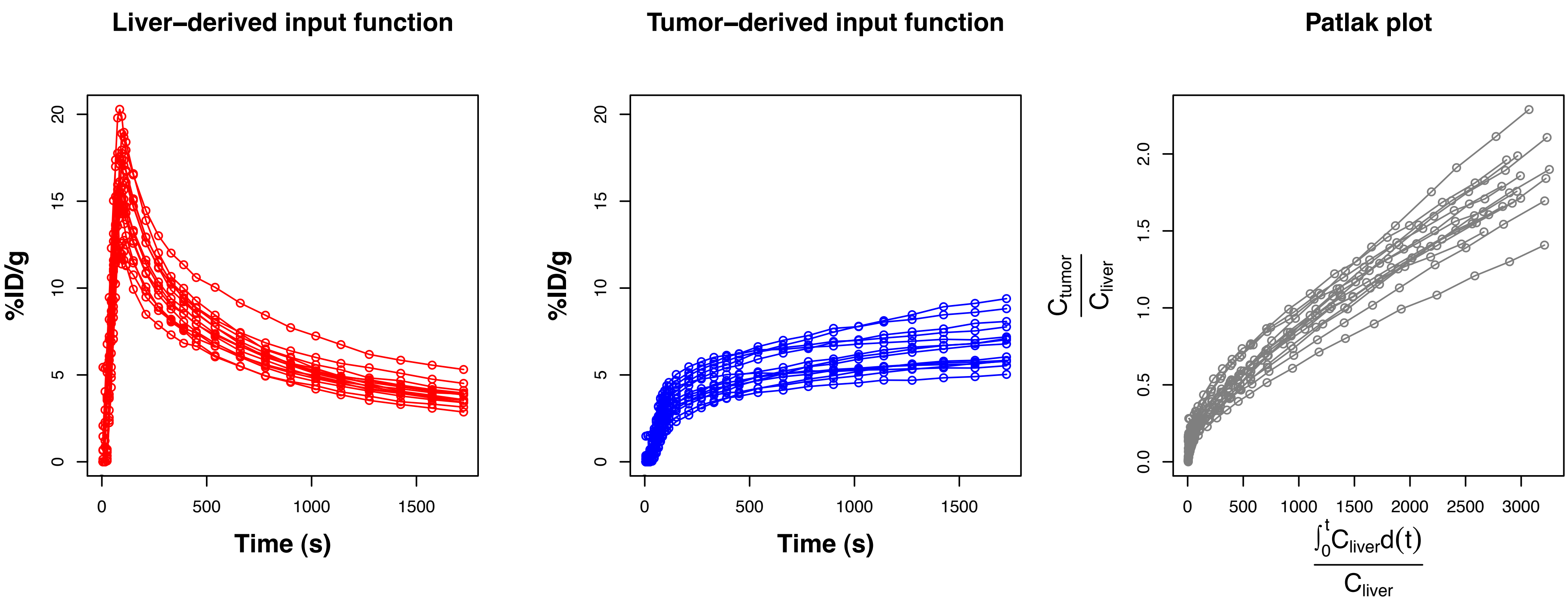

Model E. H292 lung cancer cells in C.B−17 scid mice

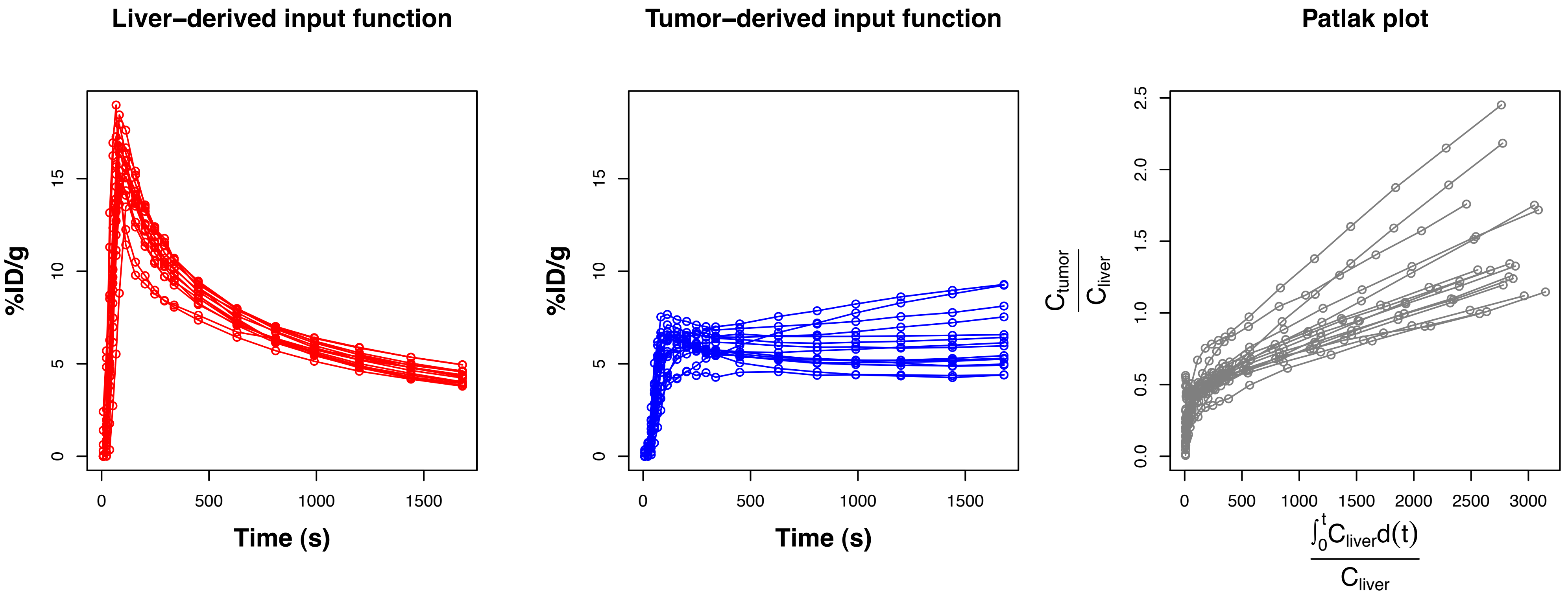

Model F. H596 lung cancer cells in huHGF transgenic mice

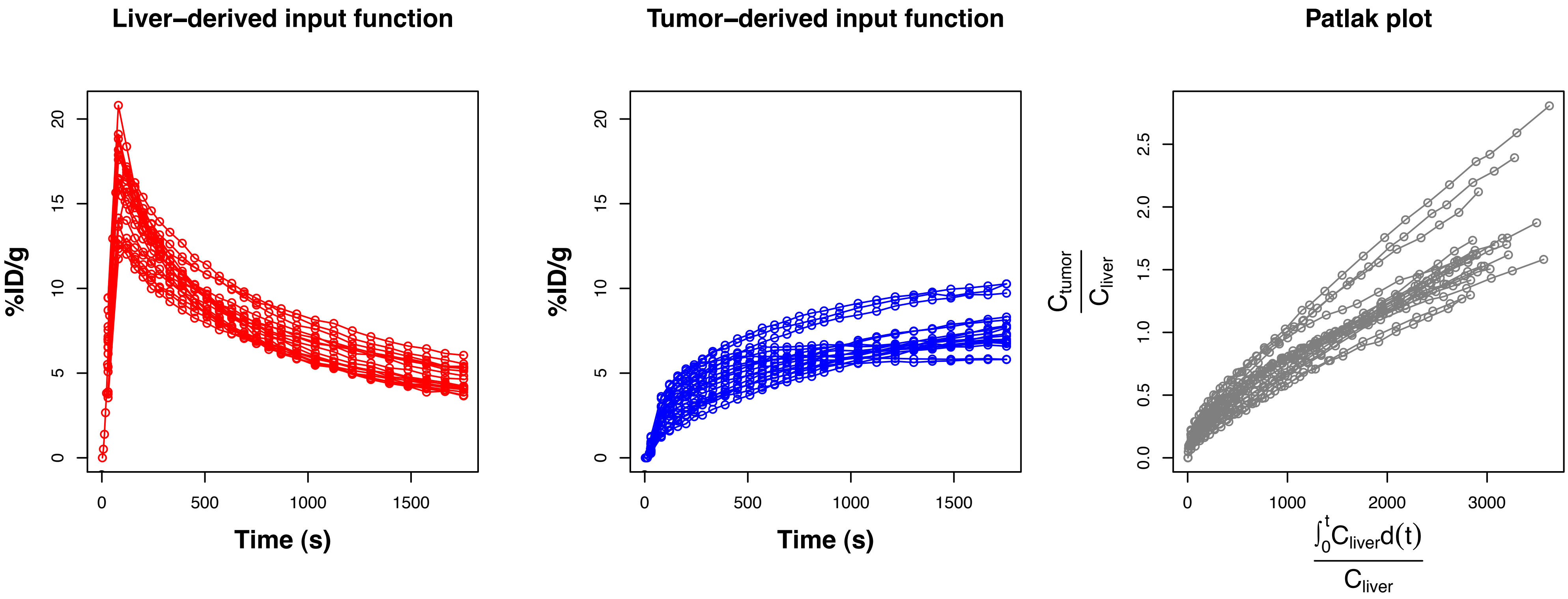

Model G. 537−MEL melanoma cells in athymic nude mice

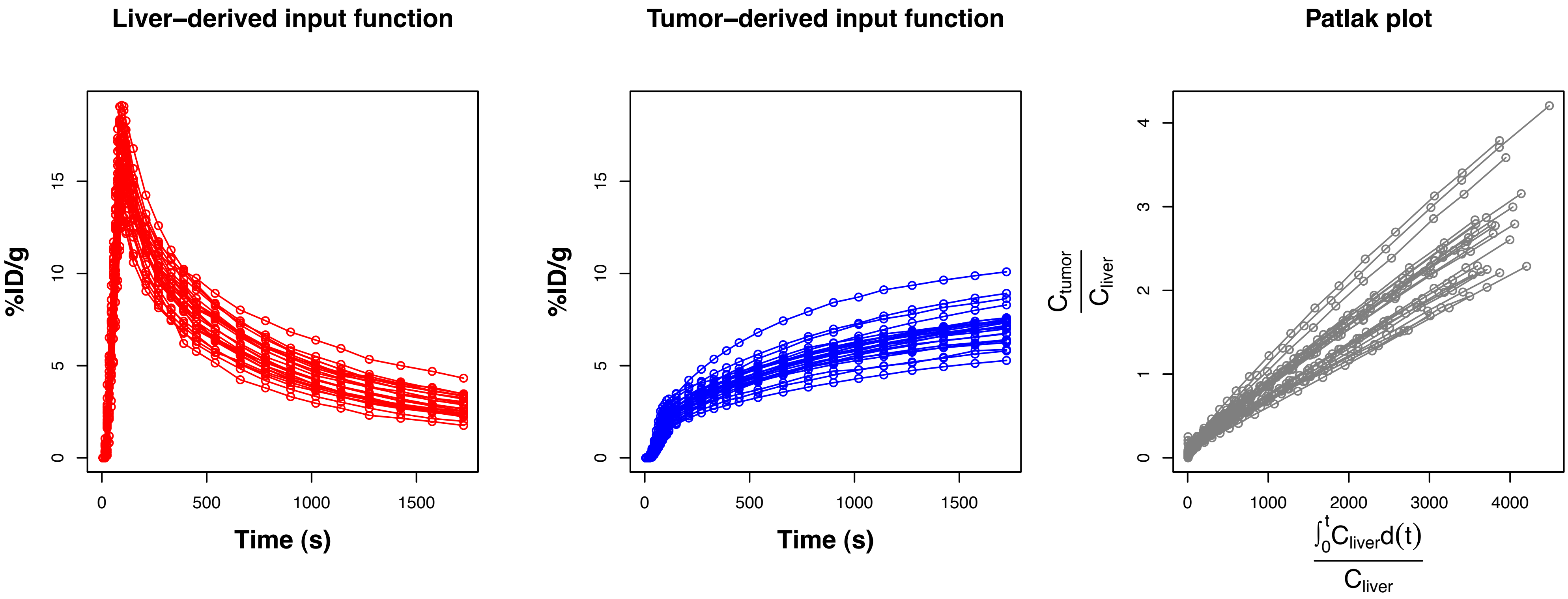

Model H. A2058 melanoma cells in athymic nude mice

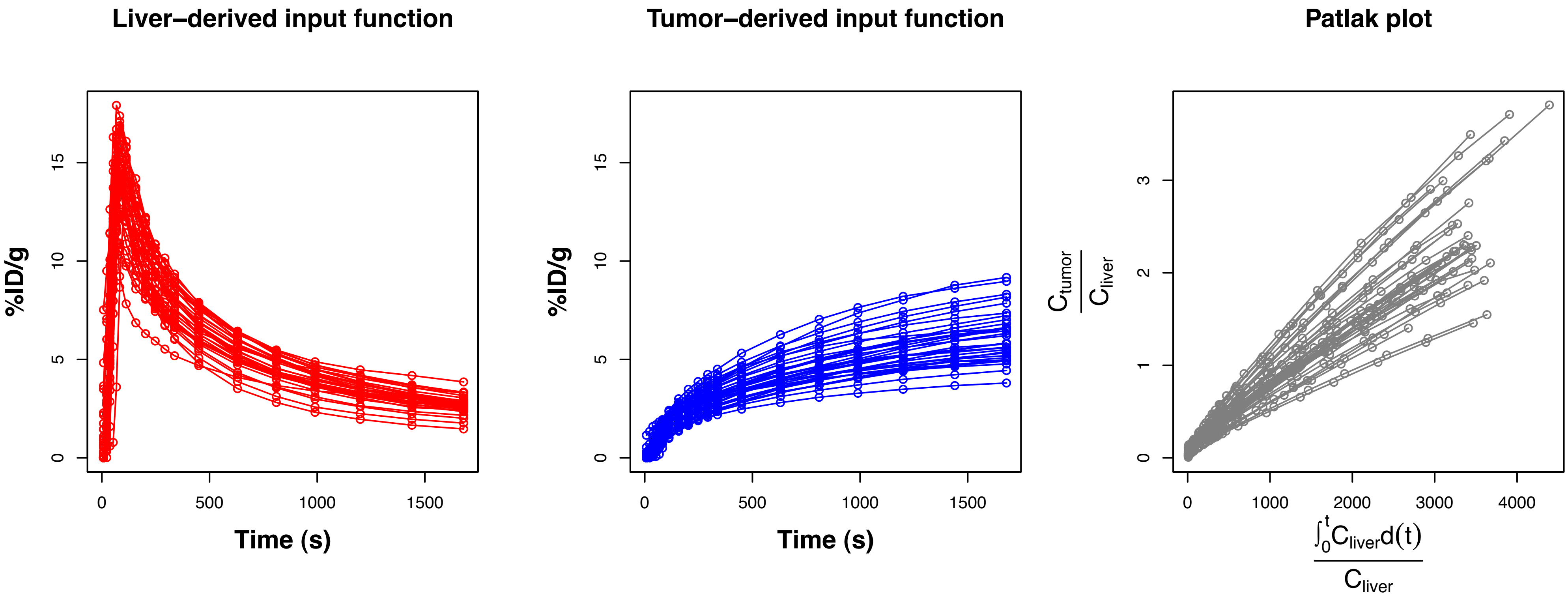

Model I. A375 melanoma cells in athymic nude mice

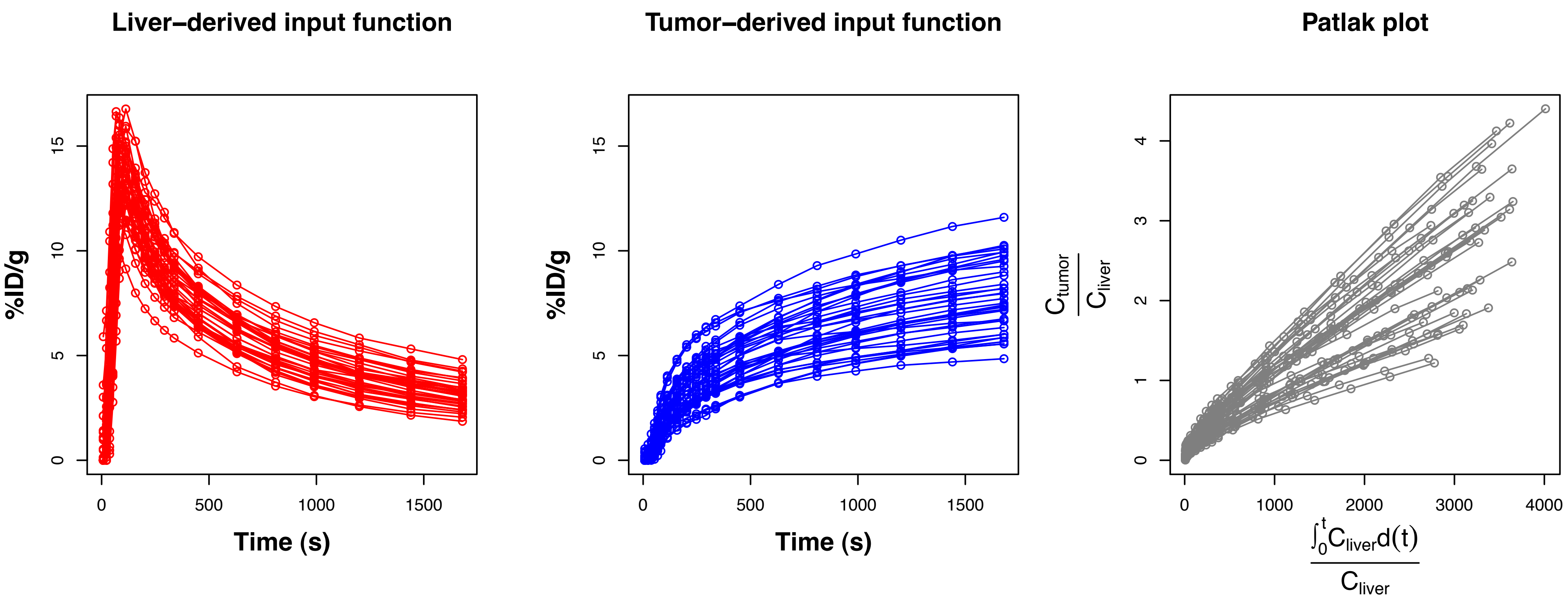

Model J. Colo205 colon cancer cells in athymic nude mice

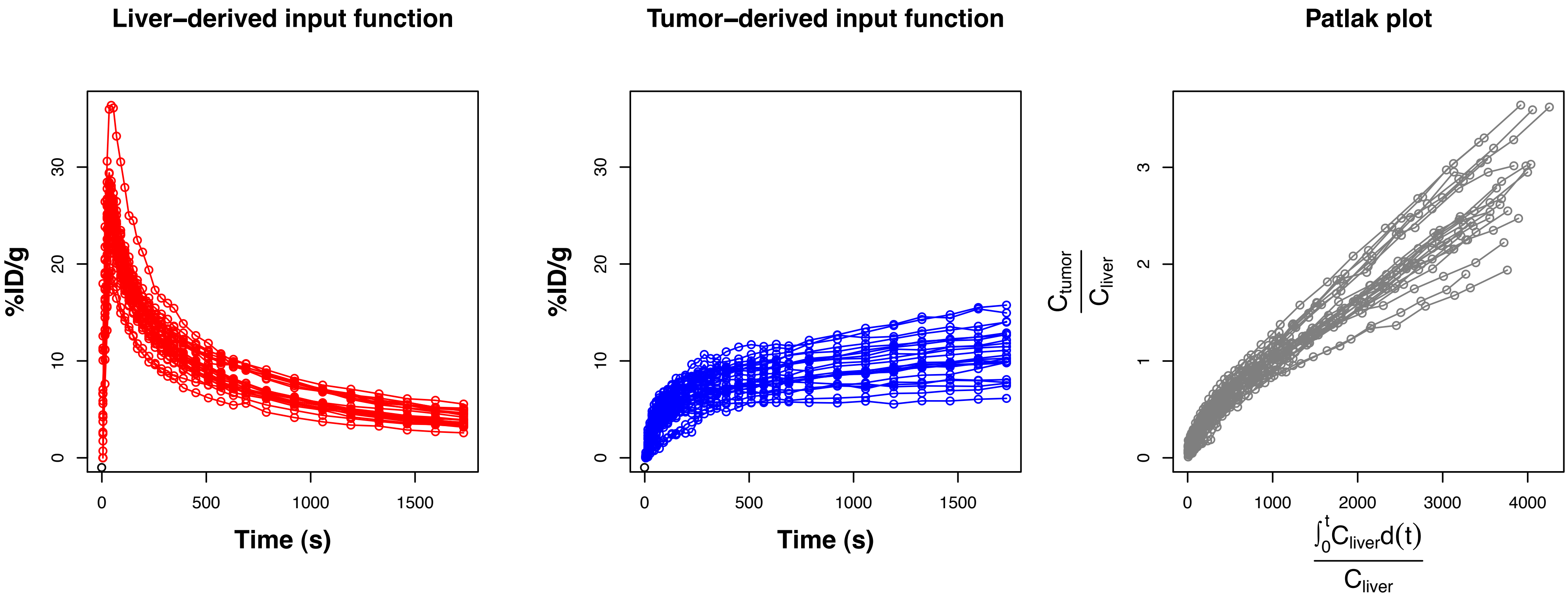

Model K. H2122 lung cancer cells in athymic nude mice

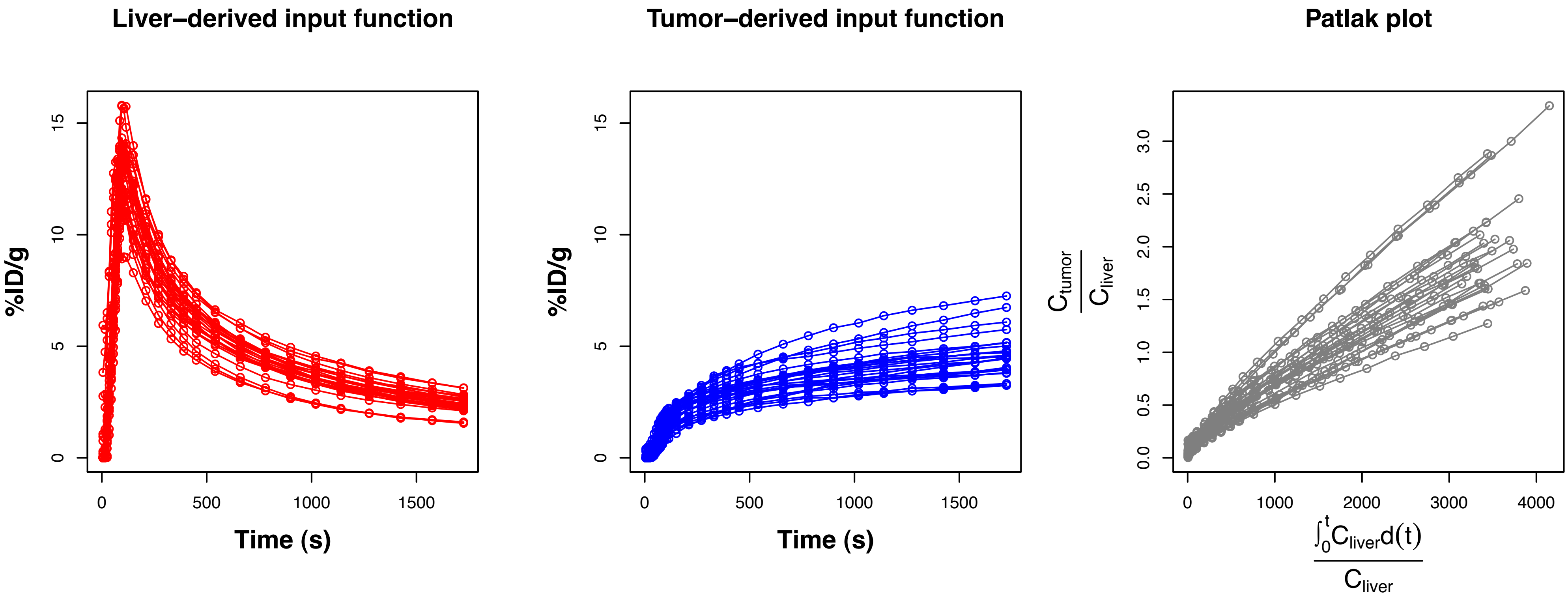

Supplement: Additional file 1 — ROI data and corresponding Patlak plots from FDG-PET scans in each of the 11 tumor models A to K discussed in the text (see Table 1). In each plot, the data from one cohort (n = 14 to 36) of essentially identical mice are superimposed. Left, in red: the liver-derived input function; center, in blue: the tumor; right, in gray: the Patlak plot. [file 2191-219X-2-6-S1.PDF]
