# Supplementary material for: Quantitation of glucose uptake in tumors by dynamic FDG-PET has less glucose bias and lower variability when adjusted for partial saturation of glucose transport
Source: EJNMMI Res. 2012 Feb 1;2:6. doi: 10.1186/2191-219X-2-6 (PMC3395842; doi:10.1186/2191-219X-2-6)
Supplement: Additional file 2 — Confidence intervals for correlations between PET metrics and blood glucose. To obtain the 95% confidence limits for Pearson's correlation coefficient (r), the Fisher transformation was applied to the sample correlation coefficients. [file 2191-219X-2-6-S2.PDF]

# Confidence intervals for correlations between PET metrics and blood glucose

To obtain the 95% confidence limits for Pearson's correlation coefficient (r), the Fisher transformation was applied to the sample correlation coefficients. The formula shown below was used in every animal model, where n= total number of animals per model.

$$95\%\ \text{C.I.} = \tanh\left(\text{arctanh}(r) \pm z_{\frac{\alpha}{2}} s.e.\right)$$

where,

$$z_{\frac{\alpha}{2}} = 1.96 \text{ standard deviations}$$

$$s.e. = \frac{1}{\sqrt{n-3}}$$

then it follows:

$$95\%\ \text{C.I.} = \tanh\left(\text{arctanh}(r) \pm \frac{1.96}{\sqrt{n-3}}\right)$$

**A.** 95 % confidence intervals of Pearson's correlation coefficients between $MRGluc^{MAX}$ and glucose.

| Model | Pearson's correlation coefficient | Lower confidence limit | Higher confidence limit |
|---|---|---|---|
| A | 0.03 | -0.25 | 0.31 |
| B | 0.25 | 0.15 | 0.35 |
| C | -0.14 | -0.32 | 0.04 |
| D | 0.4 | 0.17 | 0.58 |
| E | 0.02 | -0.11 | 0.15 |
| F | 0.06 | -0.17 | 0.28 |
| G | 0.27 | 0.06 | 0.46 |
| H | -0.16 | -0.37 | 0.07 |
| I | 0.11 | -0.21 | 0.4 |
| J | -0.1 | -0.33 | 0.15 |
| K | -0.09 | -0.31 | 0.14 |

**B.** 95 % confidence intervals of Pearson's correlation coefficients between MRGluc and glucose.

| Model | Pearson's correlation coefficient | Lower confidence limit | Higher confidence limit |
|---|---|---|---|
| A | 0.53 | 0.3 | 0.71 |
| B | 0.63 | 0.56 | 0.69 |
| C | 0.41 | 0.24 | 0.55 |
| D | 0.8 | 0.69 | 0.88 |
| E | 0.54 | 0.44 | 0.63 |
| F | 0.58 | 0.41 | 0.71 |
| G | 0.54 | 0.36 | 0.68 |
| H | 0.22 | -0.01 | 0.42 |
| I | 0.43 | 0.14 | 0.65 |
| J | 0.69 | 0.53 | 0.8 |
| K | 0.49 | 0.29 | 0.65 |

**C. 95 %** confidence intervals of Pearson's correlation coefficients between $K_i$ and glucose.

| Model | Pearson's correlation coefficient | Lower confidence limit | Higher confidence limit |
|---|---|---|---|
| A | -0.32 | -0.55 | -0.05 |
| B | -0.09 | -0.2 | 0.02 |
| C | -0.46 | -0.6 | -0.31 |
| D | -0.24 | -0.46 | 0 |
| E | -0.37 | -0.47 | -0.25 |
| F | -0.36 | -0.54 | -0.15 |
| G | 0.03 | -0.19 | 0.25 |
| H | -0.48 | -0.63 | -0.28 |
| I | -0.26 | -0.53 | 0.06 |
| J | -0.58 | -0.72 | -0.39 |
| K | -0.4 | -0.57 | -0.18 |
